# Challenges in ECG learning: a questionnaire analysis of medical students

**DOI:** 10.1186/s12909-025-08376-0

**Published:** 2025-12-02

**Authors:** Li Wang, Shouqin Sheng

**Affiliations:** 1 Department of Electrocardiographic Diagnosis, The Second Affiliated Hospital of Anhui Medical University, Hefei, Anhui China; 2 Department of Teaching Management, The Second Affiliated Hospital of Anhui Medical University, Hefei, Anhui China

**Keywords:** Electrocardiogram, Teaching, Interpretation, Questionnaire

## Abstract

**Introduction:**

Poor electrocardiogram (ECG) interpretation skills among medical students are a widespread issue globally. However, few studies have systematically investigated the practical challenges students face during the learning process. This study aimed to identify the specific obstacles encountered by medical students in learning ECG interpretation and to propose evidence-based recommendations for curricular improvement.

**Methods:**

A questionnaire survey was conducted among interns and other relevant student groups who were about to begin or had completed their ECG internship at the Second Affiliated Hospital of Anhui Medical University. A total of 309 valid, voluntarily submitted responses were collected and analyzed using descriptive statistics, difference analysis, and correlation analysis.

**Results:**

Most students agreed with statements such as "Even with solid theoretical knowledge, interpreting ECGs in practice is challenging", "Practical ECG waveforms are complex and variable, making analysis difficult", and "The abstract nature of ECG waveforms increases the threshold for learning electrocardiography". These perceptions did not differ significantly by gender, identity, level of education, or undergraduate field of study (*P* > 0.05). Correlation analysis revealed a significant association between "the absence of textual labels in ECG diagrams" and "the abstract nature of ECG waveforms may increase the difficulty of learning ECG" (*r* = 0.728), as well as between "Even with solid theoretical knowledge, ECG interpretation remains challenging" and "ECG diagrams are complex and difficult to analyze" (*r* = 0.715). Moreover, internship experience significantly reduced medical students' perceived difficulty in learning ECGs (3.99 ± 0.70 vs. 3.75 ± 0.71, *P* = 0.002).

**Conclusions:**

The primary barrier in ECG interpretation is the abstract and complex nature of waveforms. Teaching should focus on strategies to address this challenge. The proposed translation method offers a novel approach to improve learning.

**Supplementary Information:**

The online version contains supplementary material available at 10.1186/s12909-025-08376-0.

## Introduction

The insufficiency in medical students' electrocardiogram(ECG) interpretation skills has long been a focal point in cardiology education [1–3]. Numerous studies indicate that medical students face significant challenges in interpreting ECGs, which not only hampers their clinical decision-making abilities but can also adversely affect patient diagnosis and treatment [4, 5]. Current research predominantly focuses on improving general teaching methods, such as case analysis, group interactive discussions, and online resources [6–8]. Aida Bazrgar et al. [9] found that although students seemed to prefer face-to-face teaching, the blended teaching method, which combines online and face-to-face instruction, appeared to be more effective in enhancing students' ECG interpretation skills. Sunhee Lee et al. [10] developed an HTML-based webpage incorporating an ECG interpretation algorithm and found that an ECG education program incorporating this webpage helped maintain and improve nurses' interpretation skills. However, there remains a notable gap in research systematically investigating the specific difficulties students encounter when learning ECG from their own perspective. Without a clear understanding of these challenges, even the most advanced computer-assisted teaching tools risk being superficial solutions that fail to address the core learning barriers. To bridge this gap, the present study focuses on students’ experiences, employing a survey to comprehensively explore the real difficulties they face in ECG learning. By identifying these challenges, we aim to propose targeted improvements to optimize ECG education and enhance learning outcomes.

## Objects and methods

### Survey subjects and sample size

The survey was conducted between May 9, 2024 and June 19, 2024. The participants included medical undergraduates from the class of 2019 who had completed their internships at the Second Affiliated Hospital of Anhui Medical University, medical undergraduates from the class of 2020 who were about to begin their internships, and residents undergoing standardized training at the same hospital from 2021 to 2023. Participants' educational backgrounds encompassed medical undergraduates, postgraduates, and associate degree students. All participants had received formal ECG instruction during their medical education.

The initial sample size was calculated using the formula [11]:

$$n=\frac{{Z}^{2}.p.(1-p)}{{e}^{2}}$$

The adjusted sample size was calculated according to the formula [12]:

$${n}_{Adjustment}=\frac{nN}{n+N-1}$$

with a total population of 920, an expected proportion (*p*) of 0.5, an allowable error (E) of 5%, and a confidence level corresponding to a Z-value (Z) of 1.96, the required sample size was calculated to be 272. A total of 309 valid online questionnaires were received, exceeding the required sample size and meeting the survey requirements.

### Content and methodology of the survey

The questionnaire used in this study was independently developed by three ECG physicians based on their clinical teaching experience (see Supplementary Material for the full version).

A pilot test was conducted among 69 medical students to evaluate the clarity and relevance of the questionnaire items, and minor wording modifications were made accordingly. The final questionnaire consisted of two sections: the first section collected demographic information (5 items); the second section employed a 5-point Likert scale to assess students' perceptions in the dimension of "difficulties in ECG learning." This scale comprised six items, with response options ranging from "Strongly Agree" (5 points) to "Strongly Disagree" (1 point). For readability, simplified item wordings are presented in the main text, while the complete versions are provided in the supplementary materials.

Notably, in the Chinese medical education system, medical students are enrolled in specific undergraduate programs from the beginning of their training, such as clinical medicine, anesthesiology, or optometry. These majors indicate their academic focus rather than clinical licensure or professional specialization. The students identified by their major in this study were still completing their undergraduate medical education during supervised clinical rotations. In addition, Item 5 in Part I, which asks about the type of ECG learning the participants received, was used for descriptive purposes only and was not included in the main statistical analysis.

The questionnaire was collected by the staff of the teaching management department of our hospital. It was distributed via a link or Quick Response code in the WeChat groups of interns and doctors. The questionnaire survey in this study was conducted on a non-compulsory and voluntary basis, with the distribution carried out online. A total of 920 questionnaires were distributed, and 411 were returned, resulting in a response rate of 44.7%. Considering the non-incentivized nature of the online survey, this response rate falls within the expected range and did not significantly impact the representativeness of the sample. To ensure data quality, we excluded responses that showed logical inconsistencies. For example, if a respondent strongly agreed with statements indicating significant difficulty in learning or interpreting ECGs, yet strongly disagreed with the statement "When interpreting an ECG, you may still feel unsure about where to begin and how to apply your knowledge effectively", such a response was flagged as logically inconsistent. Additionally, uniformly extreme answers were excluded. Potential duplicate entries were identified and removed using IP addresses, submission timestamps, and unique user identifiers provided by the online survey platform, ensuring the independence and representativeness of the final sample. After excluding logically inconsistent or repeated questionnaires, 309 valid questionnaires were obtained, yielding an effective rate of 75.2%.

### Statistical method

The survey data were coded, entered into a database, and analyzed using SPSS 18.0. Statistical analyses included descriptive statistics, t-tests, one-way analysis of variance and Pearson correlation, with *p* < 0.05 considered statistically significant. The internal consistency of the questionnaire was assessed using Cronbach's α. Sampling adequacy for factor analysis was examined with the Kaiser–Meyer–Olkin (KMO) measure and Bartlett's test of sphericity. Exploratory factor analysis (EFA) was conducted using the principal axis factoring method to determine the underlying construct structure of the questionnaire. As this was an observational cross-sectional analysis, causal relationships cannot be inferred.

### Ethics statement

The study protocol, including the informed consent process, was reviewed and approved by the ethics committee of The Second Affiliated Hospital of Anhui Medical University (approval number: YX2024-070, May 9, 2024). The study was conducted in accordance with the principles of the Declaration of Helsinki. Participants were informed and gave their consent before starting the questionnaire. The consent process was integrated into the questionnaire introduction, which outlined the purpose of the study, the voluntary nature of participation, and the confidentiality of the data. By proceeding to complete the questionnaire, participants implicitly provided their consent.

## Results

### Reliability and validity assessment

The reliability and validity of the questionnaire were first examined. Cronbach's alpha for the overall scale was 0.872, demonstrating good internal consistency and indicating that the items measure the same underlying construct in a coherent manner. The KMO value was 0.821, exceeding the recommended threshold of 0.80, and Bartlett's test was statistically significant (*p* < 0.001). These results confirm that the data were well-suited for factor analysis and provide support for the structural validity of the questionnaire.

The communalities analysis showed that the extracted variances of all items ranged from 0.48 to 0.62, indicating that the common factor had a relatively strong explanatory power for the items and provided a solid basis for the structural validity of the scale. According to the criterion of eigenvalues greater than 1, a single factor, labeled "ECG learning difficulties," was ultimately extracted, accounting for 53.8% of the total variance. The factor matrix revealed that all items loaded above 0.69 on this factor and no cross-loadings were detected (see Table 1 and Table 2).

**Table 1:** Total variance explained

Component	Initial Eigenvalues			Extraction Sums of Squared Loadings		
	Total	% of Variance	Cumulative %	Total	% of Variance	Cumulative %
1	3.685	61.410	61.410	3.226	53.761	53.761
2	0.789	13.150	74.560			
3	0.519	8.644	83.203			
4	0.499	8.324	91.527			
5	0.267	4.448	95.975			
6	0.241	4.025	100.000			

Extraction method: Principal Axis Factoring

**Table 2:** Factor Matrix

Item	Factor 1
Q5. Practical ECG waveforms are complex and variable, making analysis difficult	0.785
Q2. There are no textual cues in ECG waveforms	0.763
Q6. It is hard to know where to start when interpreting ECGs in practice	0.726
Q4. Even with solid theoretical knowledge, interpreting ECGs in practice is challenging	0.723
Q1. The abstract nature of ECG waveforms may increase the difficulty of learning ECG	0.702
Q3. ECG content is extensive and scattered	0.695

Extraction method: Principal Axis Factoring. One factor extracted; 5 iterations required. Q1–Q6 denote the six Likert-scale items in the Part II of the questionnaire. Simplified item descriptions are used in the table for readability, and the original wording is provided in the Supplementary Material

### Demographic characteristics and overview of ECG learning

Among the 309 respondents, 172 were male (55.66%) and 137 were female (44.34%). The educational levels included 3 associate degree students (0.97%), 270 undergraduate students (87.38%), and 36 Master's students (11.65%). Regarding specialties, 182 (58.90%) majored in clinical medicine, 49 (15.86%) in other clinical fields, 31 (10.03%) in optometry, 25 (8.09%) in anesthesiology, and 22 (7.12%) in pediatrics. A total of 151 students (48.87%) had only studied the theoretical knowledge of ECG in university classrooms without participating in practical ECG training. In contrast, 158 students (51.13%) had both learned the theoretical knowledge of ECG in university classrooms and completed the internship tasks in the ECG diagnosis department at our hospital.

### Scores on ECG learning difficulties and attitudes toward teaching methods

The questionnaire included 10 items, denoted as Q1–Q6 (see Supplementary Materials for the original wording; simplified forms are presented here for readability). The respondents' mean scores for ECG learning difficulties was 3.87 ± 0.71. Items Q1–Q3 primarily reflected challenges related to theoretical knowledge learning, while items Q4–Q6 reflected difficulties in practical ECG interpretation. Among theoretical learning difficulties, "The abstract nature of ECG waveforms may increase the difficulty of learning ECG" received the highest score (3.85 ± 0.95). Within the practical interpretation difficulties, "Even with solid theoretical knowledge, interpreting ECGs in practice is challenging" had the highest score (4.05 ± 0.84). Detailed results are presented in Table 3.

**Table 3:** Medical students' attitudes toward project-related descriptions

Item	No. (%)					
	** Strongly Agree (5 points)**	**Agree (4 points)**	**Neutral (3 points)**	**Disagree (2 points)**	**Strongly Disagree (1 point)**	**Average Score (**$\overline{{\varvec{x}} }$**±s)**
Q1. The abstract nature of ECG waveforms may increase the difficulty of learning ECG	85 (27.51)	122 (39.48)	77 (24.92)	20 (6.47)	5 (1.62)	3.85±0.95
Q2. There are no textual cues in ECG waveforms	70 (22.65)	131 (42.39)	67 (21.68)	33 (10.68)	8 (2.59)	3.72 ± 1.01
Q3. ECG content is extensive and scattered	65 (21.04)	143 (46.28)	73 (23.62)	23 (7.44)	5 (1.62)	3.78 ± 0.92
Q4. Even with solid theoretical knowledge, interpreting ECGs in practice is challenging	93 (30.10)	158 (51.13)	44 (14.24)	9 (2.91)	5 (1.62)	4.05 ± 0.84
Q5. Practical ECG waveforms are complex and variable, making analysis difficult	85 (27.51)	156 (50.49)	55 (17.8)	12 (3.88)	1 (0.32)	4.01 ± 0.80
Q6. It is hard to know where to start when interpreting ECGs in practice	73 (23.62)	131 (42.39)	74 (23.95)	30 (9.71)	1 (0.32)	3.79 ± 0.92

Responses were collected using a 5-point Likert scale ranging from 1 (strongly disagree) to 5 (strongly agree). Higher scores indicate a more positive attitude

### Analysis of attitudes toward ECG learning difficulties across different groups

Statistical analyses revealed no significant differences in ECG learning difficulties across gender, identity, level of education, or undergraduate field of study (*p* > 0.05), indicating that these demographic factors did not exert a substantial influence on the study outcomes. Regarding ECG learning difficulties, students without internship experience scored significantly higher than those who had completed internships (3.99 ± 0.70 vs. 3.75 ± 0.71, *p* = 0.002). Detailed results are presented in Table 4.

**Table 4:** Evaluation scores by demographic group

Category	Groups	ECG learning difficulties
Gender	Male(*n* = 172)	3.88 ± 0.71
	Female(*n* = 137)	3.85 ± 0.72
	p	0.69
Identity	Intern(*n* = 251)	3.88 ± 0.73
	Resident(*n* = 58)	3.79 ± 0.64
	p	0.367
Level of Education	Associate degree students(*n* = 3)	3.06 ± 1.21
	Undergraduate students(*n* = 270)	3.89 ± 0.69
	Master's students(*n* = 36)	3.76 ± 0.81
	p	0.078
Undergraduate Field of Study	Clinical Medicine(*n* = 182)	3.82 ± 0.74
	Pediatrics(*n* = 22)	3.80 ± 0.62
	Other Clinical (*n* = 49)	3.81 ± 0.68
	Optometry(*n* = 31)	4.11 ± 0.65
	Anesthesiology(*n* = 25)	4.05 ± 0.63
	p	0.175
ECG Internship Experience	No(*n* = 151)	3.99 ± 0.70
	Yes(*n* = 158)	3.75 ± 0.71
	p	0.002*

Mean evaluation scores are shown for subgroups based on demographic characteristics

^*^p < 0.001 indicate statistically significant differences between groups based on ANOVA tests

### Correlation coefficient

Pearson correlation analysis was conducted for all items, showing that all correlations were positive at the *p* < 0.01 level (see Table 5), indicating consistency in reflecting both theoretical and practical challenges. The strongest correlation was observed between "There are no textual cues in ECG waveforms" and "The abstract nature of ECG waveforms may increase the difficulty of learning ECG" (*r* = 0.728). This strong association suggests a possible link between students' perception of abstractness and the absence of textual cues, although causality cannot be inferred. Another strong correlation was found between "Even with solid theoretical knowledge, interpreting ECGs in practice is challenging" and "Practical ECG waveforms are complex and variable, making analysis difficult" (*r* = 0.715), suggesting that students' perception of practical difficulties is closely associated with the inherent complexity of ECG graphs. Other correlation coefficients ranged from 0.411 to 0.623, demonstrating moderate associations across different aspects of learning difficulties.

**Table 5:** Pearson correlation analysis results for Q1-Q6

Item	Q1	Q2	Q3	Q4	Q5	Q6
Q1	1					
Q2	0.728**	1				
Q3	0.506**	0.494**	1			
Q4	0.411**	0.494**	0.537**	1		
Q5	0.458**	0.528**	0.512**	0.715**	1	
Q6	0.488**	0.537**	0.527**	0.487**	0.623**	1

**p < 0.01

## Discussion

In ECG, waveform interpretation is particularly important and constitutes a major difficulty in learning [13]. As a tool for recording the invisible electrical activity of the human heart [14], the ECG waveform is highly abstract and lacks intuitive cognitive reference for medical students. Our survey further supports this view: the majority of students agreed that "The abstract nature of ECG waveforms may increase the difficulty of learning ECG", and "abstract ECG graphics" and "lack of textual cues in ECG graphics" were significantly correlated, suggesting that students' perception of ECG abstraction may be associated with the lack of textual cues. It should be emphasized, however, that this result only reflects a statistical association between the two variables and cannot be interpreted as causal. Further stratified analysis indicated that this learning difficulty was consistent across students of different genders, statuses, educational levels, and professional backgrounds, indicating its generalizability. Based on these findings, future teaching improvements may consider transforming abstract waveforms into textual and structured information, which may reduce students' cognitive load to some extent and alleviate the learning difficulty caused by the "graphical abstraction" of ECGs.

The finding that students perceive ECG waveforms as abstract and difficult to interpret can be understood through cognitive learning theories. According to Cognitive Load Theory [15], the inherent complexity and variability of ECG patterns impose a high intrinsic cognitive load on learners, which may hinder information processing when appropriate instructional support is lacking. Furthermore, Dual Coding Theory [16] suggests that learning is enhanced when verbal and visual information are integrated. The predominance of abstract visual information in ECG learning, with limited textual or conceptual cues, may therefore restrict effective encoding and retention. These theoretical perspectives help explain why students experience persistent difficulties and highlight the need for instructional designs that reduce cognitive load and promote multimodal learning.

Meanwhile, we also found that "Even with solid theoretical knowledge, interpreting ECGs in practice is challenging" was widely acknowledged by students, suggesting that they perceive a gap between theoretical learning and practical skills. In the correlation analysis of Q1–Q6, this item was significantly correlated with "Practical ECG waveforms are complex and variable, making analysis difficult", indicating that the perceived gap between theory and practice may be associated with the complexity of ECG waveforms. This finding further supports the presence of a perceived gap between theoretical learning and practical skills, although further research is needed to explore this in depth. Notably, medical students' practical ECG internship experience was associated with a reduction in perceived difficulty in ECG interpretation. During internships, students accumulated interpretive experience by encountering more ECG cases and gradually improved their interpretive skills. Therefore, compared with students without internship experience, those with internship experience reported lower perceived difficulty in ECG interpretation. This is consistent with previous studies [17, 18]. Based on these observations, we propose a potential teaching strategy: teachers can classify ECG cases into different difficulty levels and progressively present them according to students’ learning stages, thereby implementing individualized instruction and minimizing the gap between theory and practice.

Currently, widely used teaching strategies—including online resources, blended learning, and even algorithm-based ECG education platforms—have not fundamentally addressed the core difficulties that students encounter in learning ECGs [19, 20]. Although these methods may improve teaching formats or learning pace, they often fail to address the primary cognitive challenge faced by students, namely understanding abstract graphical information.

As the main executors of teaching, teachers, with their systematic understanding of ECG principles, are well positioned to play a key role in overcoming this barrier. For example, they can develop more intuitive and comprehensible teaching materials, simplify complex waveform patterns, or construct concise conceptual frameworks to help learners understand the underlying logic of ECGs from a broader and deeper perspective.

Meanwhile, the rapid development of artificial intelligence (AI) has brought new opportunities to medical education. Increasingly innovative educational tools are emerging that may have a profound impact on teaching models. However, without targeted design or proper instructional integration, AI tools may merely replicate the shortcomings of traditional methods—providing general support without addressing the core learning difficulties. Therefore, we recommend a targeted integration of AI in instructional design: teachers act as the primary agents responsible for identifying and addressing learning difficulties, while AI serves as an auxiliary tool to present and reinforce teaching strategies. In this way, abstract and complex ECG waveforms can be gradually deconstructed into simpler, more comprehensible components, thereby reducing students' cognitive load and enhancing learning efficiency.

### Suggested strategy

**Fig. 1:**
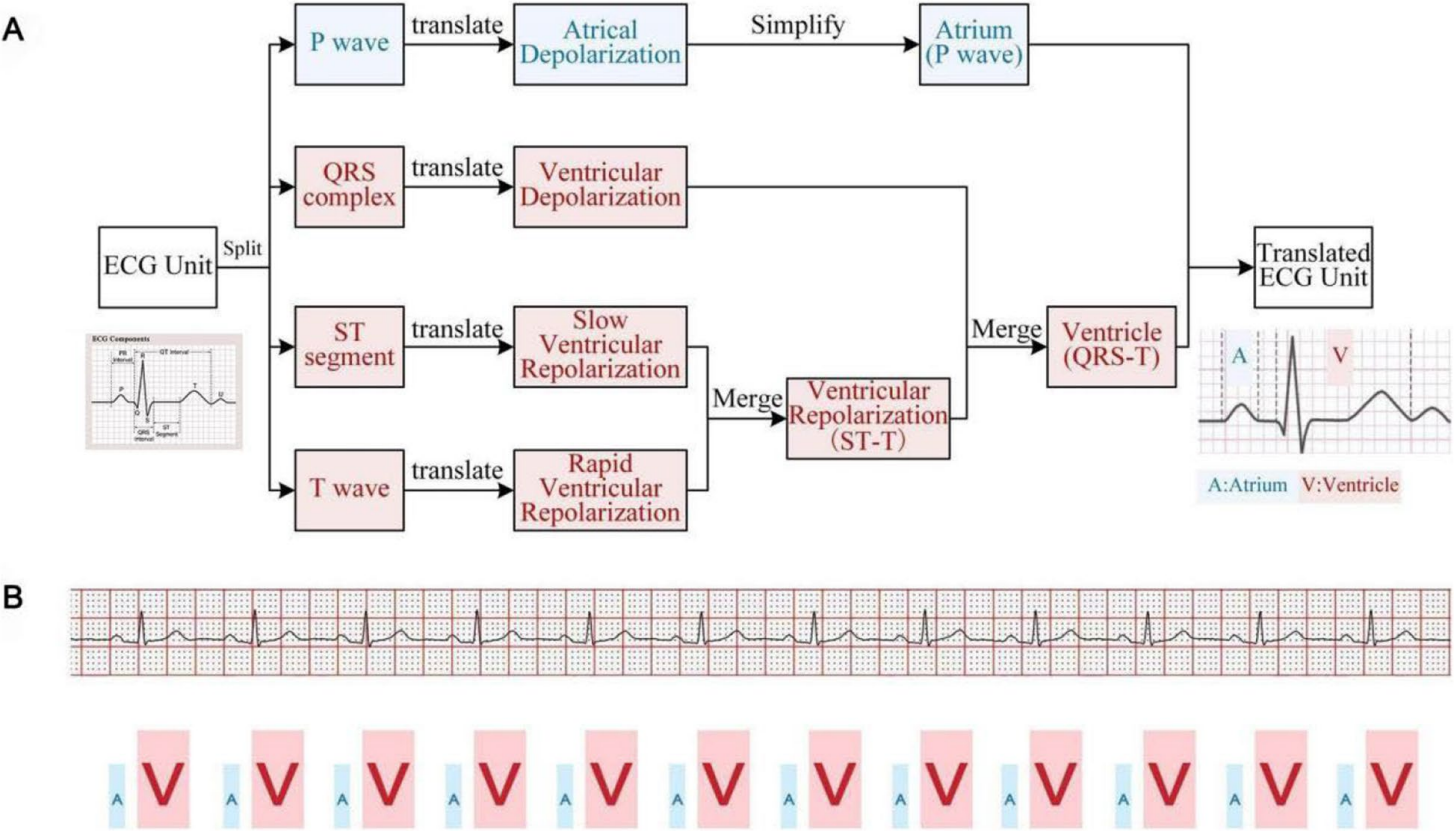
Legend: A represents the atria; V represents the ventricles. Figure **A**. Step-by-step demonstration of the ECG translation method. Figure **B**. Final representation of the ECG translation method

To address the challenges students face in interpreting abstract and complex ECG waveforms, we propose a novel instructional strategy termed the ECG Waveform Translation Method (see Figure 1A). This method aims to lower the cognitive threshold by mapping abstract waveform segments into familiar conceptual representations.

To implement this strategy, we treat the ECG waveform from a single heartbeat as an "ECG unit" comprising four standard components: P wave, QRS complex, ST segment, and T wave. These components are then conceptually transformed through a structured five-step approach. First, we begin by recognizing these four key components as the structural foundation of the ECG unit. Second, each component is interpreted based on its physiological significance: the P wave represents atrial depolarization, the QRS complex denotes ventricular depolarization, the ST segment reflects the initial phase of ventricular repolarization, and the T wave corresponds to the later, more rapid phase of ventricular repolarization. Third, the ST segment and T wave are integrated into a unified concept termed "ventricular repolarization," labeled as the ST–T segment. Fourth, the QRS complex and ST–T segment are combined to represent the complete process of ventricular excitation and recovery—commonly referred to as the QT interval. Finally, for mnemonic simplification, the P wave and QT interval are reduced to the conceptual labels "atrium" and "ventricle", respectively.

After these five steps, the abstract ECG waveform is transformed into a set of simple and familiar word symbols (see Figure 1B). This method is both intuitive and easy to remember. More importantly, it allows students to clearly recognize that continuous ECG waveforms reflect the orderly conduction of electrical signals from the atria to the ventricles, thus bridging the gap between the abstract curves and the anatomy of the heart. In this teaching strategy, the teacher continuously reinforces these ECG concepts, while the AI serves as an aid for waveform visualization, dynamic transformation display, and personalized exercise feedback, thus improving teaching efficiency and effectiveness 1.

In light of the present findings, the proposed ECG Waveform Translation Method represents an innovative pedagogical approach. By mapping abstract ECG waveform segments into familiar conceptual representations, it aims to lower the cognitive threshold for students and facilitate learning. While the method holds promising pedagogical value, it remains conceptual and has not yet been empirically tested. Therefore, it should be regarded as a theoretical proposition rather than an evidence-based instructional tool. Future studies could assess its effectiveness in enhancing students' comprehension of ECG patterns through experimental or longitudinal designs.

### Strengths and limitations

A major strength of this study lies in its focus on exploring the challenges of ECG learning from the students' own perspective—an aspect often overlooked in previous research. The results demonstrated that the primary obstacles in ECG learning stem from the abstract nature of the waveforms, the gap between theoretical knowledge and practical interpretation, and the complexity and variability of the patterns. By employing a structured Likert-scale questionnaire, this study conducted quantitative analyses including descriptive statistics, subgroup comparisons, and correlation analyses, thereby enhancing the accuracy and objectivity of the findings. Based on these results, we proposed targeted teaching strategies to address core theoretical learning barriers.

Nevertheless, several limitations should be acknowledged. First, the questionnaire was self-developed. Although factor analysis supported its construct validity, the lack of expert review and formal assessments such as Content Validity Index or Content Validity Ratio may have limited its rigor; future studies should include these procedures to strengthen content validity. Second, the consistently high agreement rates across items may reflect students' shared difficulties or be partly influenced by item wording and acquiescence bias. Positively worded statements, such as "ECG waveforms are abstract and difficult to understand" may have prompted agreement; future revisions should consider reverse-coded items and cognitive interviews to reduce bias. Third, several sampling characteristics constrain the generalizability and applicability of our findings. The study's confinement to a single institution and its 44.7% response rate limit its external validity. Specifically, the predominance of clinical medicine students means the results may not directly apply to other medical specialties or institutions with different curricula. Furthermore, the absence of non-responder analysis makes it impossible to rule out selection bias, which could affect the sample's true representativeness. Therefore, these results should be applied to broader educational contexts with caution. Finally, the cross-sectional design precludes causal inference, although correlation analyses suggested potential associations.

## Conclusion

Most students identified the abstract, complex, and variable nature of ECG waveforms as the primary challenge in ECG learning. This difficulty persisted regardless of students' gender, academic status, or educational background. Therefore, it is essential to optimize teaching content by transforming abstract concepts into concrete forms. Based on our findings, we propose innovative instructional strategies to support this goal. Educators and curriculum designers are encouraged to adopt targeted approaches that address these specific learning difficulties. Integrating theoretical instruction with hands-on application may significantly enhance students' ECG interpretation skills.

## Supplementary Information

Supplementary Material 1

## Data Availability

The datasets used and/or analysed during the current study are available from the corresponding author on reasonable request.

## References

[1] Hurst JW. Methods used to interpret the 12‐lead electrocardiogram: pattern memorization versus the use of vector concepts. Clin Cardiol. 2000;23(1):4–13. 10.1002/clc.4960230103. 10680023 10.1002/clc.4960230103 PMC6655172

[2] Kashou A, May A, DeSimone C, et al. The essential skill of ECG interpretation: how do we define and improve competency? Postgrad Med J. 2020;96(1133):125–7. 10.1136/postgradmedj-2019-137191. 31874907 10.1136/postgradmedj-2019-137191

[3] Rahimpour M, Shahbazi S, Ghafourifard M, et al. <article-title update="added">Electrocardiogram interpretation competency among emergency nurses and emergency medical service (EMS) personnel: a cross‐sectional and comparative descriptive study. Nurs Open. 2021;8(4):1712–9. 10.1002/nop2.809. 33611852 10.1002/nop2.809 PMC8186699

[4] Salerno SM, Alguire PC, Waxman HS. Training and competency evaluation for interpretation of 12-lead electrocardiograms: recommendations from the American College of Physicians. Ann Intern Med. 2003;138(9):747–50. 10.7326/0003-4819-138-9-200305060-00012. 12729430 10.7326/0003-4819-138-9-200305060-00012

[5] Cairns AW, Bond RR, Finlay DD, et al. <article-title update="added">A computer-human interaction model to improve the diagnostic accuracy and clinical decision-making during 12-lead electrocardiogram interpretation. J Biomed Inform. 2016;64:93–107. 10.1016/j.jbi.2016.09.016. 27687552 10.1016/j.jbi.2016.09.016

[6] O’Brien KE, Cannarozzi ML, Torre DM, et al. Training and assessment of ECG interpretation skills: results from the 2005 CDIM survey. Teach Learn Med. 2009;21(2):111–5. 10.1080/10401330902791255. 19330688 10.1080/10401330902791255

[7] Mahler SA, Wolcott CJ, Swoboda TK, et al. <article-title update="added">Techniques for teaching electrocardiogram interpretation: self‐directed learning is less effective than a workshop or lecture. Med Educ. 2011;45(4):347–53. 10.1111/j.1365-2923.2010.03891.x. 21401682 10.1111/j.1365-2923.2010.03891.x

[8] Nilsson M, Bolinder G, Held C, Johansson BL, Fors U, Östergren J. Evaluation of a web-based ECG-interpretation programme for undergraduate medical students. BMC Med Educ. 2008;8:25. 10.1186/1472-6920-8-25. 18430256 10.1186/1472-6920-8-25 PMC2394519

[9] Bazrgar A, Rahmanian M, Ghaedi A, et al. Face-to-face, online, or blended: which method is more effective in teaching electrocardiogram to medical students? BMC Med Educ. 2023;23(1):566. 10.1186/s12909-023-04546-0. 37559020 10.1186/s12909-023-04546-0 PMC10413712

[10] Lee S, Kim HJ, Choi Y, et al. Effectiveness of electrocardiogram interpretation education program using mixed learning methods and webpage. BMC Med Educ. 2024;24(1):1039. 10.1186/s12909-024-05960-8. 39334173 10.1186/s12909-024-05960-8 PMC11428852

[11] Lwanga SK, Lemeshow S. Sample size determination in health studies: a practical manual. Geneva: World Health Organization; 1991.

[12] Naing L, Winn T, Rusli BN. Practical issues in calculating the sample size for prevalence studies. Arch Orofac Sci. 2006;1:9–14.

[13] Simpson SA, Gilhooly KJ. Diagnostic thinking processes: evidence from a constructive interaction study of electrocardiogram (ECG) interpretation. Appl Cogn Psychol. 1997;11(6):543–54.

[14] AlGhatrif M, Lindsay J. A brief review: history to understand fundamentals of electrocardiography. J Community Hosp Intern Med Perspect. 2012;2(1):14383. 10.3402/jchimp.v2i1.14383. 10.3402/jchimp.v2i1.14383 PMC3714093 23882360

[15] Sweller J. Cognitive load during problem solving: effects on learning. Cogn Sci. 1988;12(2):257–85.

[16] Paivio A. Mental representations: a dual coding approach. New York: Oxford University Press; 1986.

[17] Hancock EW, Deal BJ, Mirvis DM, et al. AHA/ACCF/HRS recommendations for the standardization and interpretation of the electrocardiogram. Part V: electrocardiogram changes associated with cardiac chamber hypertrophy: a scientific statement from the American Heart Association Electrocardiography and Arrhythmias Committee, Council on Clinical Cardiology; the American College of Cardiology Foundation; and the Heart Rhythm Society. J Am Coll Cardiol. 2009;53(11):992–1002. 10.1016/j.jacc.2008.12.015. 19281932 10.1016/j.jacc.2008.12.015

[18] Balady GJ, Bufalino VJ, Gulati M, et al. COCATS 4 task force 3: training in electrocardiography, ambulatory electrocardiography, and exercise testing. J Am Coll Cardiol. 2015;65(17):1763–77. 10.1016/j.jacc.2015.03.021. 25777646 10.1016/j.jacc.2015.03.021

[19] Breen CJ, Kelly GP, Kernohan WG. ECG interpretation skill acquisition: a review of learning, teaching and assessment. J Electrocardiol. 2022;73:125–8. 10.1016/j.jelectrocard.2019.03.010. 31005264 10.1016/j.jelectrocard.2019.03.010

[20] Patuwo P, Wagner GS, Ajijola OA. Comparison of teaching basic electrocardiographic concepts with and without ECGSIM, an interactive program for electrocardiography. Comput Cardiol. 2007;34:61–4. 10.1109/CIC.2007.4745421.

