# Supplementary material for: Challenges in ECG learning: a questionnaire analysis of medical students
Source: BMC Med Educ. 2025 Dec 2;26:16. doi: 10.1186/s12909-025-08376-0 (PMC12777255; doi:10.1186/s12909-025-08376-0)
Supplement: Supplementary file 1 — Supplementary Material 1 [file 12909_2025_8376_MOESM1_ESM.docx]

**ECG Learning Status Survey Questionnaire**

**Participant Information and Consent Statement**

Dear Participant,

Thank you for taking the time to participate in this survey on the current status of student learning in electrocardiography (ECG). This study is designed to investigate the experiences of students in learning ECG and to identify potential learning difficulties. Your participation will contribute to a deeper understanding of the challenges encountered in ECG education and provide valuable evidence for improving teaching practices.

Please be assured that all responses will be kept strictly confidential and used solely for academic research purposes. No personally identifiable information will be collected, and participation in this survey involves no risk.

By proceeding with the questionnaire, you indicate your informed consent to participate in this study.

**Part 1: Demographic Information Survey**

1. Your Gender:

□Male

□Female

2. Your Identity:

□Intern

□Resident

1. Your Level of Education:

□Associate degree students

□Undergraduate students

□Master's students

4. Your Undergraduate Field of Study:

□Clinical Medicine

□Pediatrics

□Anesthesiology

□Optometry

□Other Clinical

5. Which forms of ECG learning have you experienced?

□ Self-directed study

□ Online ECG courses

□ Classroom-based lectures

□ Practical ECG laboratory sessions (operation and waveform interpretation)

□ Clinical internship in an ECG department

**Part 2: Survey on the Difficulty of Learning Electrocardiography**

1.You believe that the abstract nature of ECG waveforms makes them difficult to understand and raises the barrier to learning ECG.

□Strongly Agree

□Agree

□Neutral

□Disagree

□Strongly Disagree

2.ECG waveforms, which consist solely of graphic curves without textual clues, can present a significant challenge.

□Strongly Agree

□Agree

□Neutral

□Disagree

□Strongly Disagree

3.You think the ECG content to be mastered is scattered and the number of knowledge points is huge.

□Strongly Agree

□Agree

□Neutral

□Disagree

□Strongly Disagree

4.You believe that even if the theoretical aspects are well understood, correctly interpreting ECGs still presents challenges.

□Strongly Agree

□Agree

□Neutral

□Disagree

□Strongly Disagree

5.You believe that the complexity and variability of ECG patterns make graphical analysis confusing and prone to errors.

□Strongly Agree

□Agree

□Neutral

□Disagree

□Strongly Disagree

6.When interpreting an ECG, you may still feel unsure about where to begin and how to apply your knowledge effectively.

□Strongly Agree

□Agree

□Neutral

□Disagree

□Strongly Disagree
